# Supplementary material for: Shape- and Size-Controlled Palladium Nanocrystals and Their Electrocatalytic Properties in the Oxidation of Ethanol
Source: Materials (Basel). 2021 May 31;14(11):2970. doi: 10.3390/ma14112970 (PMC8197974; doi:10.3390/ma14112970)
Supplement: Supplementary file 1 [file materials-14-02970-s001.zip › materials-1195352-supplementary.pdf]

materials

MDPI

Supplementary Materials

Shape- and Size-Controlled Palladium Nanocrystals and Their Electrocatalytic Properties in the Oxidation of Ethanol

Seokhee Lee [1,†], Hyeongkyu Cho [1,†], Hyeon Jeong Kim [2], Jong Wook Hong [3,*] and Young Wook Lee [4,*]

1 Energy & Environment Division, Korea Institute of Ceramic Engineering and Technology (KICET), Jinju 52851, Korea; lsh@kicte.re.kr (S.L.); hkcho@kicet.re.kr (H.C.)
2 Department of Chemistry and Research Institute of Natural Sciences, Gyeongsang National University, Jinju 52828, Korea; jane9598@naver.com
3 Department of Chemistry, University of Ulsan, Ulsan 44610, Korea
4 Department of Education Chemistry and Research Institute of Natural Sciences, Gyeongsang National University, Jinju 52828, Korea
* Correspondence: jwhong@ulsan.ac.kr (J.W.H.); lyw2020@gnu.ac.kr (Y.W.L.)
† These authors contributed equally to this work.

Table S1. FT-IR spectra of IR peak (cm^{-1}) assignments for CTAC and CTAC-Pd NPs.

CTAC	Pd-CTAC	Assignments
3020	3011	CH_{3asym} (-N+(CH_3)$_3$)
2944	2985	CH_{3sym}(-N+(CH_3)$_3$)
2923	2939	CH_{2asym}
2854	2900	CH_{2sym}
1491	1490	$\delta(CH_3)_{asym}$
1479	1471	$(CH_2)_{def}$
1464	1453	$(CH_2)n_{sciss}$
		$\delta(N^+CH_3)_{sym}$
1406	1406	
1394	1392	
	1388	N=O

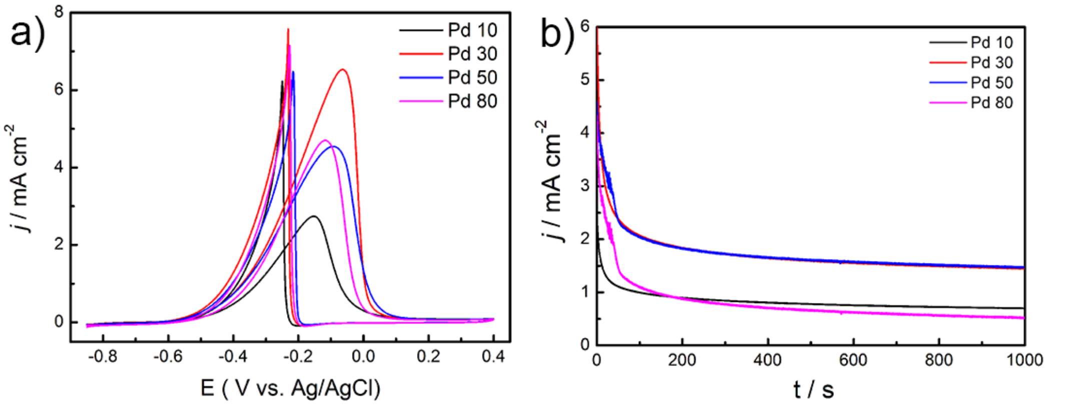

Figure S1. Cyclic voltamograms of (**a**) Specific activity for ethanol electrooxidation on different electrode in 0.5 M ethanol +0.1 M KOH with scanning rate of 50 mVs^{-1} (**b**) Chronoamperometric curves for ethanol electrooxidation at −0.1 V versus Ag/$AgNO_3$ on a Pd NPs electrode Pd10, Pd30, Pd50 and Pd80 in a 0.1 M ethanol +0.1 M KOH solution.

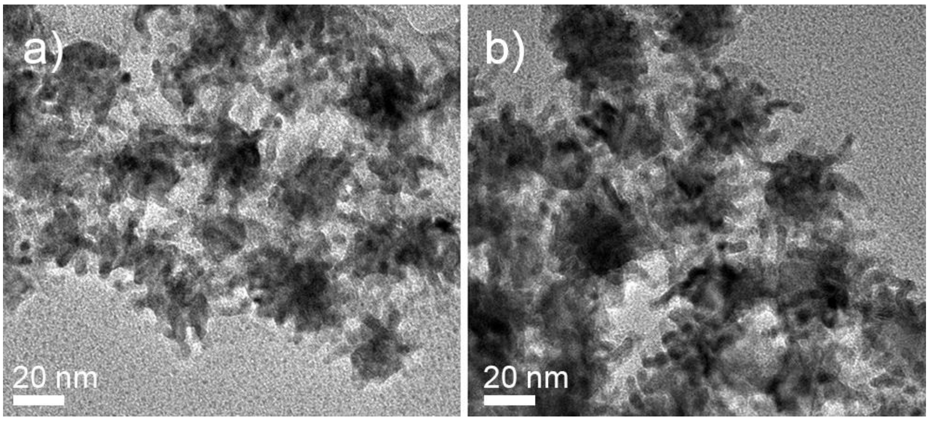

Figure S2. TEM images of Pd 30 catalysts (**a**) before and (**b**) after Chronoamperometric curves.
